# Genome Sequence of a Potential Probiotic Strain, *Lactobacillus fermentum* HFB3, Isolated from a Human Gut

**DOI:** 10.1128/genomeA.01296-15

**Published:** 2015-11-05

**Authors:** Madhu Kumari, Mohit Kumar Swarnkar, Sanjay Kumar, Anil Kumar Singh, Mahesh Gupta

**Affiliations:** a Department of Food Neutraceuticals and Quality Control, CSIR, Institute of Himalayan Bioresource Technology, Palampur, Himachal Pradesh, India; b Department of Biotechnology, CSIR, Institute of Himalayan Bioresource Technology, Palampur, Himachal Pradesh, India

## Abstract

A draft genome sequence of 2.04 Mb is reported for *Lactobacillus fermentum* HFB3, which is a lactic acid bacterium with probiotic properties. The gene-coding clusters also predicted the presence of genes responsible for probiotic characteristics.

## GENOME ANNOUNCEMENT

The human gut contains a large number of bacterial populations that play an important role in maintaining host health. Among them, lactobacilli have drawn significant attention because of their health-promoting effects that include increased resistance to infection (1), stabilization of intestinal microflora (2), augmentation of the immune system (3), cholesterol reduction (4), and cancer prevention (5). Lactobacilli are generally regarded as safe (GRAS) because of their long history of safe use in fermented foods. *Lactobacillus fermentum* is an obligatory heterofermentative bacterium belonging to the family *Lactobacillaceae* of the phylum *Firmicutes* (6). *L. fermentum* strain HFB3 is a rod-shaped, Gram-positive, and catalase-negative bacterium. Strain HFB3 was isolated from a fecal sample from a healthy tribal individual inhabiting Bundala Village of Kangra District, Himachal Pradesh (32°07′57.59″N, 76°32′21.59″E; mean salinity [msal], 1,386 M). People living in this area are adapted to local diets and fermented foods. Therefore, the identification of probiotics associated with the gut of people in this population holds promise for the isolation of new probiotic strains. The 16S rRNA gene sequencing of HFB3 revealed that it shares 99.54% similarity with *L. fermentum* CECT 562^T^. *L. fermentum* strain HFB3 has probiotic attributes, such as tolerance to bile salts, simulated gastric and intestinal juices, antimicrobial activity against pathogenic microorganisms, and cell surface hydrophobicity.

For genome sequencing of this strain, the genomic DNA was isolated using the phenol chloroform-isoamyl alcohol extraction procedure from 24-h-old *L. fermentum* HFB3 culture in MRS broth incubated at 37°C. The quantity and quality of the genomic DNA were determined by NanoDrop 2000 UV-vis spectrophotometer (Thermo Scientific, USA) and Qubit 2.0 fluorometer (Invitrogen, USA). The genomic DNA was sheared using Covaris g-tubes and checked with a Bioanalyzer DNA 12000 Chip (Agilent Technologies, USA). The genomic DNA library with 10-kb DNA insert was prepared using the PacBio SMRTbell library preparation kit version 1.0, as per the manufacturer’s instructions. The SMRTbell library was sequenced on a PacBio RS II system on two single-molecule real-time (SMRT) cells employing P5 polymerase and C3 chemistry with a 180-min movie. The SMRT cells produced 641,963,641 bases generated through 74,272 reads, with an *N*_50_ size of 18,143 bp and mean subread length of 8,643 bp (7). The filtered subreads were assembled *de novo* using CLC Genomics Workbench version 8.0.3 (Qiagen, Aarhus A/S). Functional annotation was performed on the Rapid Annotations using Subsystems Technology (RAST) server. The complete circular genome was 2,043,356 bp, with 51.77% G+C content. RAST predicted 2,512 protein-coding genes and 75 RNA genes, assigned through 75 RAST subsystem categories (8). Based on RAST annotation, strain HFB3 is closely related to *L. fermentum* IFO 3956 (score, 542) and *L. fermentum* ATCC 14931 (score, 530).

Genome analysis of *L. fermentum* HFB3 identified genes that possess characteristics of bile salt hydrolysis (choloylglycine hydrolase), cell adhesion (fibronectin-binding and cell surface proteins), antioxidative properties (thioredoxin, thioredoxin reductase, and glutathione reductase), exopolysaccharide production (glycosyltransferase), bacteriocin production (colicin V production protein), and folate biosynthesis (dihydrofolate synthase). In addition, alpha-galactosidase and beta-galactosidase biosynthetic genes were also identified that contribute to the digestion and absorption of lactose. Therefore, *L. fermentum* strain HFB3 can be used as a potential candidate for probiotic food products.

### Nucleotide sequence accession numbers.

This whole-genome shotgun project has been deposited at DDBJ/EMBL/GenBank under the accession no. LJFJ00000000. The version described in this paper is version LJFJ01000000.
